# COPD, diabetes, lymphopenia, and increased LDH cause higher mortality in hospitalized COVID‐19 patients

**DOI:** 10.1002/mco2.243

**Published:** 2023-03-20

**Authors:** Guilherme C. Frison, Sainan V. da Cunha, Lucas Q. Antoniazzi, Paulo H. K. de Oliveira, Joao P. S. Oliveira, Vinicius F. Cury, Clara Fontanari, Enrico E. Moretto, Verônica A. Oliveira, Renato Seligman

**Affiliations:** ^1^ School of Medicine Federal University of Rio Grande do Sul Porto Alegre Rio Grande do Sul Brazil; ^2^ Internal Medicine Service Hospital de Clinicas de Porto Alegre Porto Alegre Rio Grande do Sul Brazil

Dear Editor,

The clinical presentation of COVID‐19 has a wide spectrum, from asymptomatic to severe cases that require hospitalization for ventilatory support. To optimize care, it is necessary to identify which patients have the greatest potential for a serious outcome. Several comorbidities and biomarkers have been studied to assess the prognosis of patients with severe acute respiratory syndrome (SARS) CoV‐2 infection.

The aim of the present study was to evaluate risk factors for mortality in patients with Covid‐19 admitted with SARS, to Hospital de Clinicas de Porto Alegre, Brazil, a teaching general hospital. We conducted a cross‐sectional study, nested to a retrospective observational cohort that comprised a sample of 936 patients aged 18 years or older with COVID‐19, confirmed by positive real time polymerase chain reaction (RT‐PCR) or antigen testing and SARS, included from March 2020 to May 2021, to assess risk factors for pulmonary thromboembolism (PTE) (Figure 1A). We included all patients submitted to pulmonary angiotomography due to worsening of oxygenation and suspicion of PTE. Clinical and laboratorial data were collected in the electronic medical records of all patients. A total of 680 patients were admitted to intensive care unit (ICU) and 256 to a dedicated COVID‐19 area to receive oxygen support, including high‐flow nasal oxygen therapy and noninvasive ventilation. From these 936 patients, 285 did not perform laboratory tests included in research protocol and were not enrolled for analysis. Patients were divided into survivors and nonsurvivors. Lymphopenia was defined as lower than 690 cells/μL, and lactate dehydrogenase (LDH), defined elevated when superior to 482 U/L (Figure 1C). We modeled D‐dimers into a categorical variable, taking a positive result to be equal or greater than five times the reference value, considering the elevation of D‐dimers caused by COVID‐19 inflammation previously reported.^1^ Materials and Methods are detailed in supplementary materials.

**FIGURE 1:**
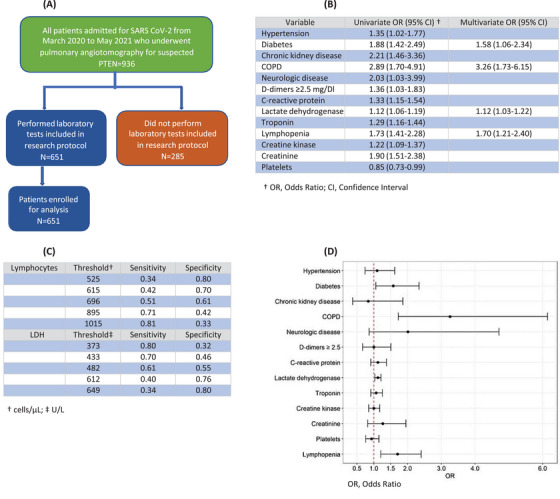
(A) Flowchart of the study cohort. (B) Odds ratio for mortality in COVID‐19 patients. Univariate and multivariate analyses. (C) Receiver operating characteristic curve analysis of laboratory values taken on admission at different cutoffs in predicting mortality of COVID‐19 patients. (D) Forrest plot ratio for mortality in COVID‐19 Patients.

As expected, there was significant increase in mortality among patients admitted to ICU. Hypertension, diabetes, chronic kidney disease, dialysis, neurologic diseases, and chronic obstructive pulmonary disease (COPD) were also significant predictors in univariate analysis. There was no difference in mortality between genders, cerebrovascular disease, heart disease, asthma, lung diseases other than asthma and COPD, tobacco consumption, liver cirrhosis, non‐active malignancy, immunosuppression, previous organ transplant and HIV carriers.

Results of various laboratory tests were different between survivors and nonsurvivors. The biomarkers C‐reactive protein (CRP), LDH, troponin, creatine phosphokinase (CPK), creatinine, lactate, hemoglobin, platelets, neutrophils, lymphocytes, neutrophil/lymphocyte rate and D‐dimers ≥2.5 mg/dL were associated with mortality (*p* < 0.05). Bilirubin, urea, monocytes, fibrinogen, and activated partial thromboplastin time were not predictors. All observed variables are listed in Supplementary Materials (Table S1).

Our results demonstrated that COPD, diabetes, lymphopenia, and LDH were significant independent predictors of mortality in multivariate logistic regression model, even after adjusting for hypertension, chronic kidney disease, neurologic diseases, CRP, troponin, CPK, creatinine, hemoglobin, platelets, neutrophils, and D‐dimers ≥2.5 mg/dL (Figure 1B,D).

We observed in univariate analysis that increased levels of LDH (odds ratio (OR) 1.12; CI 1.06–1.19) and lymphopenia (OR 1.73; CI 1.41–2.28) corresponded, respectively, to a 12% and 73% increase in mortality. Multivariate logistic regression model showed similar values, presenting 12% increase in mortality (OR: 1.12; 95% CI: 1.03–1.25) for LDH and 70% (OR 1.70; CI 1.21–2.40) for lymphopenia, demonstrating the practical prognostic relevance of LDH and lymphocyte count, both isolated or in association with other markers.

Our data showed increased LDH levels on hospital admission can predict mortality in COVID‐19 patients. These results are comparable to a recent meta‐analysis that found a six‐fold increase in mortality associated with elevated LDH. On aerobic conditions human body cells convert pyruvate to acetyl‐CoA, which enters the citric acid cycle and produces adenosine triphosphate (ATP). However, under anaerobic conditions, such as tissue hypoxia provoked by COVID‐19, the enzyme LDH promotes a deviation of this mechanism towards the production of lactate through pyruvate.^2^

We found lower absolute lymphocyte count (ALC) was associated with increased COVID‐19 mortality. This association remained significant in multivariate analysis. Lymphopenia is consistently described to be independently associated with adverse clinical outcomes in COVID‐19, namely ICU admission, the use of mechanical ventilation and in‐hospital mortality. Although other studies used different ALC cutoff values, we appraised 696 lymphocytes as the most accurate cutoff value. A possible explanation for the high mortality in low lymphocyte count COVID‐19 patients may be due to an increased cellular exhaustion mediated by NKG2A+ receptors. It is well known that cytotoxic T lymphocytes (CTL) and natural killer (NK) cells play an essential role in controlling viral infection. In severely COVID‐19 ill patients, NKG2A+ expression was upregulated on NK cells and CTLs, correlating with a reduced ability to produce CD107a, Interferon‐γ (IFN‐γ), Interleukin‐2 (IL‐2), granzyme B, and Tumor Necrosis Factor‐α (TNF‐α), resulting in disease progression.^3^

Consistent with studies that identified diabetes a risk factor, our results showed an OR 1.88 (1.42–2.49) in univariate and 1.58 (95% CI 1.06–2.34) in multivariate analysis for mortality. During the H1N1 infection outbreak, the OR for ICU admission among hospitalized patients with diabetes was four times of those without it. Moreover, a known history of diabetes was attributed to be an independent predictor of death in the SARS‐COV1 outbreak. Hypotheses to justify the relationship between these two entities have already been explored. For instance, angiotensin‐converting enzyme (ACE2) works as a receptor for SARS‐CoV‐2. High ACE2 levels are often described in diabetic patients.^4^ Diabetes mellitus inhibits neutrophil chemotaxis, phagocytosis, and intracellular killing of microbes, diminishes T cell function, and lowers innate and adaptive immunity.

Finally, we found COPD a significant independent predictor of mortality, with OR of 2.89 (1.70–4.91) in univariate and 3.26 (1.73–6.15) in multivariate analysis. It is known that acute worsening of COPD has its main trigger in viral infections, including seasonal coronaviruses. Several studies that demonstrated COPD as independent predictor of mortality in patients with COVID‐19 support this association. Increased ACE‐2 expression in COPD seems to be one of the mechanisms involved in greater severity of COVID‐19.^5^ In addition, viral conditions can cause exacerbation of systemic inflammation in COPD patients and the associated cytokine storm leads to acute respiratory distress syndrome. These mechanisms, with lower baseline lung function, anatomic abnormalities and immune dysfunction found in COPD patients, seem to be the main explanations for the greater severity.

One of the limitations of this study is that the stage of chronic lung disease severity was not evaluated. Moreover, we only evaluated lymphocyte count and LDH on the day of hospital admission, not observing the kinetics throughout the treatment. Furthermore, the severity of cases included in our sample does not correspond to all patients with Covid‐19. Cases that did not require hospitalization were not included and for these our results should not be extrapolated. However, our study has strengths. The large sample size is one of the advantages of our study, with individualized review of the medical records conducted by the team of researchers. Moreover, the automation of our laboratory, integrated with the electronic medical record of patients, guarantees fidelity of results, avoiding transcription errors.

Much remains to be understood about Covid‐19. Evidence is being researched, with controversial results on certain topics. Our study provides additional data about the association of lower lymphocytes, increased LDH, COPD, diabetes mellitus to the risk of death in hospitalized COVID‐2019 patients. These biomarkers may serve as prognostic tools, highlighting the need for intensive treatment regimens for patients at risk of severe disease. These results suggest further studies to confirm our findings in other COVID‐19 settings and to investigate other management options for these patients.

## AUTHOR CONTRIBUTIONS

RS conceived the protocol and drafted the manuscript. GCF, SVC, LQA, PHKO, JPSO, VFC, CF, EEM, and VAO collected the literatures, extracted, and analyzed the data, and revised the manuscript drafts. All authors contributed to the final revision of manuscript drafts and approved this manuscript for publication.

## FUNDING INFORMATION

The authors received no specific funding for this work.

## CONFLICT OF INTEREST STATEMENT

The authors declare that they have no conflict of interest.

## ETHICS STATEMENT

This study was approved by the Hospital de Clinicas de Porto Alegre Research Ethics Committee. Patients’ informed consent was waived due to its retrospective nature. Ethics approval number: 3.850.336.

## Supporting information

Supporting Information

## Data Availability

The data supporting this study are available from the corresponding author upon reasonable request.
