# Supplementary material for: COPD, diabetes, lymphopenia, and increased LDH cause higher mortality in hospitalized COVID‐19 patients
Source: MedComm (2020). 2023 Mar 20;4(2):e243. doi: 10.1002/mco2.243 (PMC10026558; doi:10.1002/mco2.243)
Supplement: Supplementary file 1 — Supporting Information [file MCO2-4-e243-s001.docx]

COPD, Diabetes, Lymphopenia and increased LDH cause higher mortality in hospitalized COVID-19 patients.

Guilherme C. Frison1, Sainan V. da Cunha1, Lucas Q. Antoniazzi1, Paulo H.K. de Oliveira1, Joao P. S. Oliveira1, Vinicius F. Cury1, Clara Fontanari1, Enrico E. Moretto1, Verônica A. Oliveira1, Renato Seligman1, 2

1 School of Medicine, Federal University of Rio Grande do Sul, Porto Alegre, Brazil.

2 Internal Medicine Service, Hospital de Clinicas de Porto Alegre, Brazil.

**SUPPLEMENTARY MATERIALS**

MATERIALS AND METHODS

This cross-sectional study was nested to an retrospective observational study that comprised a sample of 936 patients aged 18 years or older with COVID-19 and severe acute respiratory syndrome (SARS) included from March 2020 to May 2021 to assess risk factors for pulmonary thromboembolism (PTE). All patients were submitted to pulmonary angiotomography because there was worsening of oxygenation and suspicion of PTE. Our database was approved by the Hospital Research Ethics Committee. Patients informed consent was waived due to its retrospective nature.

The electronic medical records of all patients with COVID-19 were reviewed. SARS from COVID-19 was defined as a patient with a positive result in RT-PCR (real-time reverse transcriptase-polymerase chain reaction) or antigen testing (immunochromatography); at least two of the signs and symptoms - sudden onset fever, chills, headache, cough, coryza or rhinorrhoea, sore throat, anosmia or ageusia; and who develops dyspnea, a feeling of heaviness or pressure in the chest, oxygen saturation <95% or cyanosis. General clinical data were collected on demographic characteristics, medical history, laboratory tests, and outcomes during hospitalization.

A total of 680 patients were admitted to ICU and 256 were admitted to an isolation area dedicated to Covid-19 care to receive oxygen support, including high-flow nasal oxygen therapy and noninvasive ventilation. Patients who needed Invasive mechanical ventilation, vasoactive support or continuous renal replacement therapy were admitted to ICU. From these 936 patients, 285 did not perform laboratory tests included in research protocol and were not enrolled for analysis

Patients were divided into survivors and non-survivors subgroups. A descriptive analysis of the characteristics of both groups was performed. Descriptive data were expressed as frequencies (n and %) for categorical data, median and percentiles 25 and 75 for continuous data.

We modeled the quantitative variable D-dimers into a categorical independent variable, taking a positive result to be equal or greater than 5 times the normal of our laboratory, considering the elevation of D-dimers caused by COVID-19 inflammation previously reported.

The results of LDH dosage and lymphocyte count showed asymmetric distribution and required logarithmic transformation for statistical analysis.

Categorical variables were analyzed by 2-sided Pearson chi-square and continuous variables were analyzed by 2-sided Fisher exact test. Variables were submitted to univariable logistic models. Statistically significant variables with p<0.05 were included in a multivariable regression model. All basic statistical assumptions for these models are met and all analyses were performed using available-case analysis.

Statistical analyses were performed using the Statistical Package for the Social Sciences, version 18.0® (Chicago, EUA).

Table S1. Clinical and Laboratory Characteristics of Covid-19 Patients According to Outcome †

| Variable | survivor ‡ | non-survivor ‡ | *p* |
| --- | --- | --- | --- |
| Male | 327 (53.7) | 193 (59.0) | 0.610 |
| Age | 55 (44-65) | 63 (53-70) | 0.000 |
| ICU admission | 373 (61.2) | 307 (93.9) | 0.000 |
| Hypertension | 336 (55.2) | 204 (62.4) | 0.037 |
| Diabetes | 169 (27.8) | 137 (41.9) | 0.000 |
| Chronic kidney disease | 48 (7.9) | 52 (15.9) | 0.000 |
| Cerebrovascular disease | 31 (5.1) | 21 (6.4) | 0.454 |
| Cardiopathy | 68 (11.2) | 50 (15.3) | 0.079 |
| COPD | 25 (4.1) | 36 (11.0 | 0.000 |
| Asthma | 40 (6.6) | 18 (5.5) | 0.572 |
| Cirrhosis | 10 (1.6) | 4 (1.2) | 0.781 |
| Neurologic disease | 17 (2.8) | 18 (5.5) | 0.046 |
| Malignancy | 42 (6.9) | 28 (8.6) | 0.364 |
| CRP | 141.6 (96.1-203.9) | 165.10 (97.25-241.85) | 0.000 |
| LDH | 510 (377-694) | 578 (438-779) | 0.000 |
| Troponin | 10.0 (10.0-21.6) | 13.7 (10.0-53.7) | 0.008 |
| CPK | 111.0 (54.0-325.0) | 145.0 (74.0-445.0) | 0.001 |
| Creatinine | 0.97 (0.79-1.32) | 1.17 (0.87-1.72) | 0.001 |
| Lactate | 1.30 (1.00-1.60) | 1.53 (1.10-2.03) | 0.002 |
| Hemoglobin | 12.8 (11.8-14.1) | 12.7 (11.6-14.1) | 0.013 |
| Lymphocytes | 780 (530-1120) | 580 (445-905) | 0.004 |
| Neutrophil/Lymphocyte Rate | 8.67 (9.11) | 11.53 (12.52) | 0.000 |
| Fibrinogen | 646 (553-760) | 609 (533-732) | 0.140 |
| D-dimers ≥2.5 mg/dL | 1.15 (0.60-2.45) | 1.70 (0.90-4.85) | 0.046 |

† Fisher exact test for continuous variables and Pearson Chi Square for categorical variables, 2-sided significance, 936 patients.

‡ Number of cases (%) for categorical variables, median (IQR) for continuous variables.
